# TRIM14 Inhibition Suppresses Microglial Polarization and Pyroptosis Through the NF-κB/NLRP3 Pathway to Enhance Spinal Cord Injury Repair

**DOI:** 10.1155/mi/5053685

**Published:** 2025-11-09

**Authors:** Xin Lin, Yuan Xia, Xiu Yang, Peng Niu, Hui Wang, Weihua Liu, Jianghu Huang, Feiyue Lin

**Affiliations:** ^1^Shengli Clinical Medical College of Fujian Medical University, Fuzhou 350000, China; ^2^Department of Musculoskeletal Oncology, Clinical Oncology School of Fujian Medical University, Fujian Cancer Hospital, Fuzhou 350000, China; ^3^Spinal Ward, Fuzong Clinical Medical College of Fujian Medical University, The 900th Hospital of PLA Joint Logistic Support Force, Fuzhou 350000, China; ^4^Department of Nephrology, Fujian Provincial Hospital, Fuzhou University Affiliated Provincial Hospital, Fuzhou 350000, China; ^5^Department of Orthopedics, Fujian Provincial Hospital, Fuzhou University Affiliated Provincial Hospital, Fuzhou 350000, China

**Keywords:** M1 microglia, NF-κB, pyroptosis, spinal cord injury, TRIM14

## Abstract

Spinal cord injury (SCI) triggers severe neuroinflammation, impeding recovery. While microglial M1 polarization and pyroptosis are key drivers, their upstream regulators are incompletely understood. This study investigated the role of the ubiquitin ligase tripartite motif-containing protein 14 (TRIM14) in regulating neuroinflammation following SCI. Using rat SCI models and BV2 microglia exposed to lipopolysaccharide (LPS), we assessed TRIM14 expression and its functional impact via knockdown and overexpression, alongside pharmacological neurofilament (NF)-κB inhibition (pyrrolidine dithiocarbamate [PDTC]). TRIM14 was upregulated in injured spinal cords and microglia, associated with injury severity. TRIM14 knockdown in microglia stabilized IκBα by inhibiting its ubiquitination, thereby suppressing NF-κB activation, M1 polarization, and NLRP3-mediated pyroptosis. Conversely, TRIM14 overexpression exacerbated inflammation, effects markedly reversed by PDTC. In SCI rats, intralesional AAV-CRISPR/CasRx-mediated TRIM14 silencing significantly attenuated neuroinflammation and neuronal apoptosis, enhanced axonal regeneration, and improved locomotor function. Mechanistically, TRIM14 knockdown suppressed NF-κB/NLRP3 signaling, promoting a prorepair microenvironment. These results identify TRIM14 as a critical regulator of microglial activation and pyroptosis post-SCI, suggesting its therapeutic targeting could be a viable strategy to promote neural repair.

## 1. Introduction

Spinal cord injury (SCI) triggers a complex cascade of secondary pathological events, with neuroinflammation playing a pivotal role in exacerbating tissue damage and hindering functional recovery [1, 2]. Microglia, the resident immune cells of the central nervous system (CNS), rapidly activate post-SCI and exhibit dual roles: the proinflammatory M1 phenotype exacerbates neuronal death through cytokine storms, while the anti-inflammatory M2 phenotype supports tissue repair [3, 4]. However, sustained M1 polarization dominates the chronic phase of SCI, driving persistent inflammation and creating a hostile microenvironment for neural regeneration [5]. Compounding this issue, pyroptosis—a lytic proinflammatory cell death mediated by gasdermin D (GSDMD) and NLRP3 inflammasome activation—has recently emerged as a critical amplifier of neuroinflammation in SCI models [6]. Despite advances in understanding microglial dynamics, the molecular regulators coordinating these processes remain poorly characterized, limiting therapeutic development.

The neurofilament (NF)-κB pathway, a master regulator of inflammation, governs both M1 polarization and pyroptosis by inducing pro-inflammatory cytokines (TNF-α and IL-1β) and NLRP3 expression [7–9]. Mechanistically, IκBα degradation enables NF-κB nuclear translocation, while subsequent NLRP3 inflammasome assembly facilitates caspase-1 activation and GSDMD cleavage—a hallmark of pyroptosis [10, 11]. Current studies have demonstrated that tripartite motif-containing protein 14 (TRIM14), an E3 ubiquitin ligase adaptor, regulates the NF-κB signaling pathway in various pathophysiological processes, including tumorigenesis, inflammation, and immune responses [12–14]. However, its role in neuroinflammation and SCI pathophysiology remains unexplored. Given the critical need to disrupt the NF-κB/NLRP3 axis in SCI therapy, we hypothesized that TRIM14 serves as a molecular hub linking microglial activation to pyroptotic cell death, thereby impeding neural repair.

Using integrated in vitro and in vivo approaches, we systematically investigated TRIM14's regulatory mechanisms in SCI pathogenesis. Our data reveal that TRIM14 expression escalates in injured spinal cords and lipopolysaccharide (LPS)-stimulated microglia, correlating with NF-κB activation and NLRP3 inflammasome assembly. Through loss- and gain-of-function experiments, we demonstrate that TRIM14 knockdown suppresses IκBα ubiquitination, thereby inhibiting NF-κB nuclear translocation and downstream NLRP3-dependent pyroptosis. Crucially, AAV-mediated TRIM14 silencing attenuated neuroinflammation, reduced lesion cavity formation, and enhanced axonal regeneration in rats, accompanied by significant motor function recovery. These findings establish TRIM14 as a novel regulator of the NF-κB/NLRP3 axis in SCI and identify it as a promising therapeutic target for modulating microglial responses to favor neural repair.

This study bridges critical knowledge gaps by identifying TRIM14 as a key driver of microglial M1 polarization and pyroptosis via IκBα/NF-κB signaling, demonstrating the therapeutic potential of TRIM14 inhibition in rebalancing neuroinflammation, and providing mechanistic insights into NLRP3 inflammasome regulation in SCI microenvironments. By elucidating this previously unrecognized TRIM14-NF-κB-NLRP3 axis, our work advances the conceptual framework for developing precision interventions against secondary SCI damage.

## 2. Materials and Methods

### 2.1. Cell Culture

The microglial BV2 cell line (a mouse-derived microglial cell line), primary mouse spinal cord microglia, and primary mouse spinal cord neurons used in this study were obtained from Procell (Wuhan, China). All cell types were cultured in their respective complete growth media (purchased from Procell) at 37°C in a humidified atmosphere with 5% CO_2_. The culture medium was replaced every 2 days.

### 2.2. Cell Transfection and Different Treatments

To investigate the role of TRIM14 in microglial M1 polarization and pyroptosis, BV2 cells were transfected with either TRIM14-targeting siRNA (siRNA-TRIM14) or a TRIM14-overexpressing plasmid (both synthesized by GenePharma, Shanghai, China) using Lipofectamine 2000 (Thermo Fisher Scientific, MA, USA) according to the manufacturer's protocol. After 48 h of incubation, transfection efficiency was verified by quantitative polymerase chain reaction (qPCR). For M1 polarization induction, transfected cells were stimulated with 100 ng/mL LPS (Beyotime, Shanghai, China) for 24 h. The 24 h LPS stimulation time point was selected based on established protocols from previous literature to effectively induce microglial inflammatory responses [15]. Pyroptosis was subsequently induced by additional treatment with 5 mM adenosine triphosphate (ATP; Beyotime) for 1 h. To examine TRIM14's involvement in NF-κB signaling, some transfected BV2 cells were pretreated with 100 μM pyrrolidine dithiocarbamate (PDTC; NF-κB inhibitor; Beyotime) prior to LPS or LPS/ATP stimulation.

### 2.3. Transwell Coculture System

Neuronal death and A1 astrocyte polarization induced by neuroinflammation were examined using a Transwell coculture system as previously described [16]. Briefly, BV2 cells (5 × 10^4^ cells/well) were seeded on Transwell inserts (0.4 μm pore size; Corning, NY, USA) and placed above a monolayer of primary neurons/microglia in 24-well plates for a 24 h coculture period.

### 2.4. Establishment of SCI Model and AAV Administration

Adult female Sprague–Dawley rats (200–250 g; SLAC Laboratory Animal Co., Ltd., Shanghai, China) were anesthetized with 3% isoflurane. After dissecting the paraspinal muscles, a T10 laminectomy was performed. SCI was induced using a New York University impactor device by dropping a 10 g weight from a 50 mm height onto the exposed dura, as previously described [17]. To knockdown TRIM14, we employed the CasRx technology to design and construct the expression vectors, which were then packaged with AAV (Hanyi Biotech, Guangzhou, China). The sgRNA sequences used were: sgRNA1: 5′-GCCGACGTCGCTGCTGTTTCTCGAGTCGG-3′; sgRNA2: 5′-CGGACGTCGTCGACTTGTCCCAACCTGTGG-3′. Immediately postinjury, viral vectors were microinjected using a glass micropipette (100 μm tip diameter) connected to a 10 μL Hamilton syringe and Micro Syringe Pump Controller (World Precision Instruments, FL, USA). Four injections (1 μL each; 4 × 10^9^ vg/site) were administered at 1 mm depth around the lesion epicenter (200 nL/min). The needle remained in situ for 2 min postinjection to prevent backflow. After surgery, all animals received penicillin and analgesics for 3 consecutive days, and the bladders were manually voided twice daily. All animals received subcutaneous injections of penicillin (50,000 IU/kg) and buprenorphine analgesia (0.05 mg/kg every 12 h) for 3 consecutive days postsurgery, with manual bladder expression performed twice daily until spontaneous voiding function recovered (typically 7–10 days). Body temperature was maintained at 37°C using a heating pad during recovery from anesthesia, and all procedures were conducted under aseptic conditions. All experimental animal protocols involving animal care, breeding, and surgical procedures were approved by the Institutional Animal Care and Use Committee (IACUC) of Fujian Provincial Hospital Affiliated to Fuzhou University (Approval No. IACUC-FPH-SL-20240412 [0250]).

### 2.5. RNA Extraction and qPCR Analysis

Total RNA was extracted from spinal cord tissues and BV2 cells using TRIzol reagent (Invitrogen, USA). mRNA expression levels were quantified by SYBR Green-based qPCR (Takara, Dalian, China) following established methodologie [15]. GAPDH served as the endogenous control. Gene expression data were analyzed using the 2^−ΔΔCt^ method.

### 2.6. Western Blot Analysis

Protein expression was analyzed by western blotting. Spinal cord tissues, BV2 cells, astrocytes, and neurons were homogenized in RIPA lysis buffer (Thermo Fisher Scientific). Protein concentrations were determined using a BCA assay, and 25 μg of total protein per sample was separated by 10% SDS–PAGE. Separated proteins were transferred to PVDF membranes (Thermo Fisher Scientific) using standard protocols. Membranes were blocked with 5% skim milk for 1 h at room temperature, then incubated overnight at 4°C with the following primary antibodies: anti-TRIM14 (PA908056, 1:1000, CUSABIO), anti-p65 (8242, 1:1000, Cell Signaling Technology), anti-phospho-p65 (3033, 1:1000, CST), anti-IκBα (4812, 1:1000, CST), anti-phospho-IκBα (2859, 1:1000, CST), anti-iNOS (ab283655, 1:1000, Abcam), anti-NLRP3 (ab283819, 1:1000, Abcam), anti-N-GSDMD (ER1901-37, 1:1000, HUABIO), anti-ASC (ab309497, 1:1000, Abcam), anti-cleaved caspase-1 (GTX03738, 1:1000, GeneTex), anti-C3 (ab97462, 1:1000, Abcam), anti-Bax (ab182733, 1:2000, Abcam), anti-Bcl-2 (ab196495, 1:1000, Abcam), and anti-NF (2837S, 1:1000, CST). After washing, membranes were incubated with HRP-conjugated secondary antibodies (1:5000) for 1 h at room temperature. Protein bands were visualized using ECL substrate (SEVEN, PA, USA) and quantified with ImageJ software. GAPDH (ab245355, 1:10,000, Abcam), histone H3 (ab1791, 1:1000, Abcam), and α-tubulin (ab7291, 1:10,000, Abcam) served as loading controls.

### 2.7. Cell Viability Assay

Cell viability was assessed using a CCK-8 kit (Beyotime) according to the manufacturer's protocol. Following 24 h of neuron-BV2 cell coculture, 10 μL of CCK-8 solution was added to each well of the 96-well plate. After 2 h incubation at 37°C, absorbance was measured at 450 nm using a microplate reader (Multiskan GO, Thermo Fisher Scientific).

### 2.8. ELISA Analysis

Following transfection with siTRIM14 or oeTRIM14 and subsequent LPS stimulation (100 ng/mL, 24 h) to induce M1 polarization, BV2 cell culture supernatants were collected. TNF-α and IL-1β levels were quantified using commercial ELISA kits (Proteintech, USA) according to the manufacturer's protocol. Absorbance was measured at 450 nm with a reference wavelength of 570 nm using a microplate reader (Multiskan GO, Thermo Fisher Scientific).

### 2.9. Immunofluorescence Staining

Tissue sections or cultured cells were incubated overnight at 4°C with the following primary antibodies: anti-p65 (8242, 1:200, CST), anti-NLRP3 (ab283819, 1:200, Abcam), anti-N-GSDMD (ER1901-37, 1:200, HUABIO), anti-C3 (ab97462, 1:200, Abcam), anti-iNOS (ab283655, 1:200, Abcam), anti-Iba-1 (ab283346, 1:200, Abcam), anti-NF (2837S, 1:200, CST), and anti-glial fibrillary acidic protein (GFAP) (60190-1-Ig, 1:200, Proteintech). After PBS washing, samples were incubated for 2 h at room temperature in the dark with secondary antibodies: goat anti-mouse IgG (H + L) Coralite 594 (SA00013-3, 1:500, Proteintech) and goat anti-rabbit IgG (H + L) FITC (SA00003-2, 1:500, Proteintech). Nuclei were counterstained with DAPI (1 μg/mL, Beyotime) for 5 min. Fluorescence images were acquired using a DMi8 fluorescence microscope (Leica, Wetzlar, Germany) equipped with a DFC9000 sCMOS camera and 20x/0.75 NA objective lens. Image analysis was performed using ImageJ software (v1.53, NIH, USA). For quantification, iNOS^+^Iba-1^+^ and Iba-1^+^ cells were counted in three randomly selected fields from the injury epicenter per sample.

### 2.10. Co-Immunoprecipitation (Co-IP) Assay

Cell lysates containing 500 μg total protein were precleared with Protein A/G Agarose for 1 h at 4°C. The supernatants were then incubated for 1 h at 4°C with 1 μg of the following antibodies: anti-TRIM14 (PA908056, CUSABIO), anti-IκBα (4812, CST), or normal rabbit IgG (sc-69786, Santa Cruz Biotechnology) as negative control. Immune complexes were captured by incubation with Protein A/G Plus Agarose (20423, Thermo Fisher Scientific) for 3 h at 4°C with gentle rotation. After three washes with ice-cold lysis buffer (containing 150 mM NaCl and 0.1% NP-40), the bound proteins were eluted in 2x Laemmli buffer at 95°C for 5 min. Immunoprecipitated proteins were resolved by SDS–PAGE and analyzed by western blotting as described above.

### 2.11. Calcein AM–Propidium Iodide (PI) Staining

Following experimental treatments, BV2 cells were incubated with 2 μM calcein-AM (green fluorescent live-cell marker) and 1.5 μM PI (red fluorescent dead-cell stain; Beyotime) in serum-free medium for 30 min at 37°C in the dark, according to the manufacturer's protocol. Cellular morphology and fluorescence were visualized using a DMi8 inverted fluorescence microscope (Leica, Germany) equipped with FITC (ex/em 490/525 nm) and TRITC (ex/em 557/576 nm) filter sets. Images were captured using a DFC9000 sCMOS camera. PI-positive cells were counted in three randomly selected fields per sample using ImageJ software (v1.53, NIH, USA) with the cell counter plugin, and the percentage of PI^+^ cells was calculated as: (number of PI^+^ cells/total number of cells) × 100%.

### 2.12. Apoptosis Detection by Flow Cytometry

Neuronal apoptosis was analyzed using an Annexin V-FITC/PI Apoptosis Detection Kit (Thermo Fisher Scientific) according to the manufacturer's protocol. Briefly, treated neurons were collected by gentle centrifugation (200 × *g*, 5 min), resuspended in 1x binding buffer, and stained with Annexin V-FITC (5 μL/test) and PI (10 μg/mL) for 15 min at room temperature in the dark. Samples were immediately analyzed on a FACSCalibur flow cytometer (BD Biosciences, San Jose, CA, USA) equipped with a 488 nm laser. Apoptotic cells were defined as Annexin V^+^PI^−^ (early apoptosis) and Annexin V^+^PI^+^ (late apoptosis) populations, with data analyzed using FlowJo software (v10.8, BD Biosciences). Viable cells (Annexin V^−^PI^−^) and necrotic cells (Annexin V^−^PI^+^) were excluded from apoptosis rates.

### 2.13. TUNEL Staining

Transverse spinal cord sections (5 μm thickness) were obtained from rostral segments located 5 mm from the injury epicenter using a cryostat (Leica CM1950). Tissue sections were processed with the In Situ Cell Death Detection Kit (Roche, Basel, Switzerland) following the manufacturer's protocol, with DAPI counterstaining (1 μg/mL, 5 min) for nuclear visualization. Fluorescence images were acquired using a DMi8 inverted fluorescence microscope (Leica, Germany).

### 2.14. Immunohistochemistry (IHC)

Transverse spinal cord sections (5 mm rostral to the injury epicenter) were processed for IHC. After deparaffinization in xylene and rehydration through graded ethanol series, antigen retrieval was performed in citrate buffer (pH 6.0) at 95°C for 20 min. Sections were blocked with 5% normal goat serum for 1 h at room temperature, then incubated with rabbit anti-neuronal nucle (NeuN) primary antibody (24307S, 1:200, CST) for 2 h at 37°C, followed by HRP-conjugated goat anti-rabbit IgG secondary antibody (A0208, 1:500, Beyotime) for 1 h at room temperature. DAB chromogenic reaction was developed using a DAB Substrate Kit (DA1010, Beyotime) with hematoxylin counterstaining. For quantification, three randomly selected sections per rat were imaged under a DM2000 LED microscope (Leica, Germany). NeuN^+^ neurons in the anterior horn were counted using ImageJ software (v1.53, NIH, USA).

### 2.15. Nissl Staining

Transverse spinal cord sections (5 mm rostral to the injury epicenter) were processed for Nissl staining. After deparaffinization in xylene and rehydration through a graded ethanol series (100%, 95%, and 80%), sections were stained with 0.1% cresyl violet solution (C0117, Beyotime) at 56°C for 1.5 h. Sections were then differentiated in 95% ethanol, dehydrated through an ascending alcohol series (95% and 100%), cleared in xylene, and mounted with neutral balsam (G8590, Solarbio, Beijing, China). For quantification, three randomly selected sections per animal were imaged under a DM2000 LED microscope (Leica, Germany). Nissl^+^ neurons in the anterior horn were counted using ImageJ software (v1.53, NIH, USA), with cells exhibiting distinct nucleoli and cytoplasmic Nissl bodies considered viable.

### 2.16. Lesion Identification by HE Staining

At 28 days post-SCI, rats were deeply anesthetized with 3% isoflurane and transcardially perfused with 0.9% saline followed by 4% paraformaldehyde (PFA) in 0.1 M phosphate buffer (pH 7.4). A 10 mm spinal cord segment centered on the injury epicenter was dissected and postfixed in 4% PFA for 24 h at 4°C. Tissues were dehydrated through graded ethanol series, cleared in xylene, and embedded in paraffin. Serial transverse sections (5 μm thickness) through the lesion epicenter were stained with hematoxylin (5 min) and eosin (2 min) using standard protocols. Every third section was imaged using a DM2000 LED microscope (Leica, Germany). Lesion cavity areas were quantified by tracing the non-tissue regions in ImageJ software (v1.53, NIH, USA).

### 2.17. Functional Recovery Assessment

Hindlimb motor function was evaluated using the Basso–Beattie–Bresnahan (BBB) locomotor rating scale (0 = complete paralysis to 21 = normal gait). During 5 min open-field tests at post-injury days 1, 3, 7, 10, 14, 17, 21, 24, and 28, animals were video-recorded under standardized conditions (200 cm × 200 cm arena with nonslip floor). Two independent investigators blinded to experimental groups scored each session, with final BBB scores representing the mean of both assessments. Interrater reliability was confirmed by intraclass correlation coefficient (ICC > 0.85).

### 2.18. Statistical Analysis

The normality of the data was tested using the Shapiro–Wilk test in IBM SPSS Statistics 26.0 (IBM Corp., Armonk, NY, USA). Normally distributed data were analyzed by two-tailed Student's *t*-test for two-group comparisons or one-way analysis of variance (ANOVA) with Tukey's honestly significant difference (HSD) post hoc test for multigroup comparisons. Non-normally distributed data were analyzed using the Mann–Whitney *U* test for two-group comparisons or the Kruskal–Wallis test with Dunn's post hoc correction for multigroup comparisons. No data points were excluded as outliers, and all statistical tests were two-sided, with statistical significance set at *p* < 0.05. Effect sizes (Cohen's *⁣*^*∗*^d^*⁣*^*∗*^^ for *t*-tests and η^2^ for ANOVA) were reported where applicable.

## 3. Results

### 3.1. Expression of TRIM14 in Rat Spinal Cord and BV2 Cells

**Figure 1:**
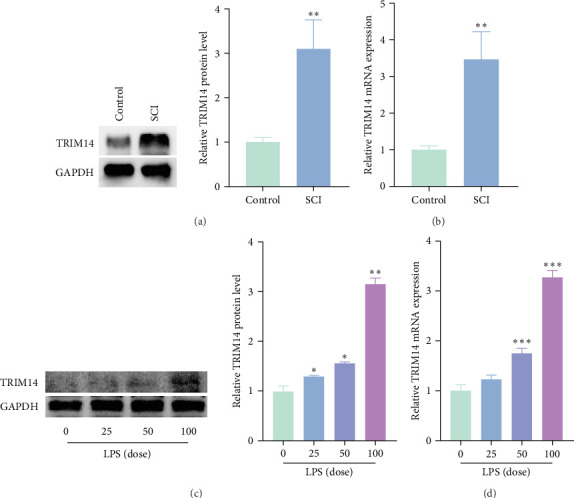
Expression of TRIM14 in damaged rat spinal cord tissues and LPS-treated BV2 cells. TRIM14 protein levels (A) and mRNA expression (B) were significantly increased at 3 days post-SCI (*n* = 3). BV2 cells were treated with various concentrations of LPS (ng/mL) for 24 h, followed by western blot (C) and RT-qPCR (D) analysis of TRIM14 (*n* = 3). Data represent mean ± SD. *⁣*^*∗*^*p* < 0.05, *⁣*^*∗∗*^*p* < 0.01, and *⁣*^*∗∗∗*^*p* < 0.001 vs. untreated control group.

As shown in Figure 1A, B, both protein and mRNA levels of TRIM14 were significantly upregulated in spinal cord tissues at 3 days postinjury (dpi), suggesting its potential role in molecular regulation after SCI. In BV2 microglial cells, TRIM14 expression exhibited a dose-dependent increase upon LPS stimulation (Figure 1C, D). The elevated expression of TRIM14 correlated with higher LPS concentrations (0–100 ng/mL), indicating its involvement in inflammatory responses in microglia.

### 3.2. TRIM14 Activates the NF-κB Pathway, Interacts With IκBα, and Promotes IκBα Ubiquitination in BV2 Cells

**Figure 2:**
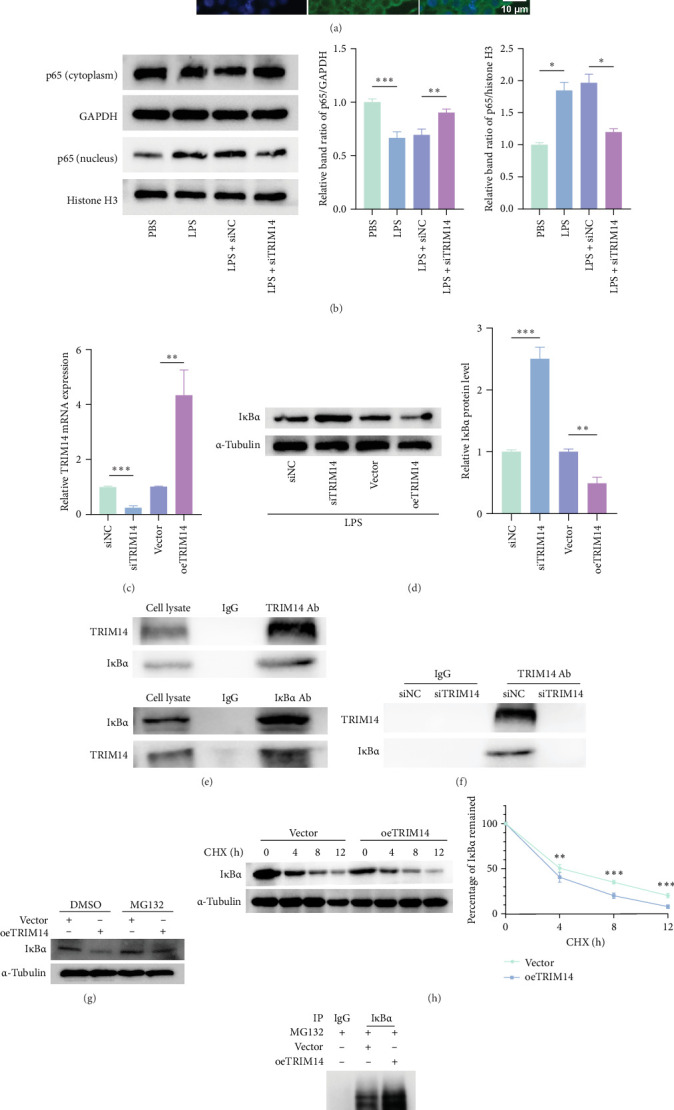
TRIM14 activates the NF-κB pathway, interacts with IκBα, and promotes IκBα ubiquitination in BV2 cells. BV2 cells were transfected with siTRIM14 and stimulated with LPS (100 ng/mL). (A) Nuclear translocation of p65 subunit was assessed by immunocytochemistry. BV2 cells were labeled with anti-p65 antibodies (green) and DAPI (blue). Representative images were obtained from three independent experiments (scale bar: 10 μm). (B) Nuclear and cytoplasmic p65 levels were measured by Western blotting, with GAPDH (cytoplasmic) and histone H3 (nuclear) as loading controls (*n* = 3). (C) Validation of TRIM14 knockdown and overexpression efficiency in BV2 cells by qRT-PCR (*n* = 3). (D) After TRIM14 knockdown or overexpression in BV2 cells followed by LPS treatment, IκBα protein levels were analyzed by Western blot (*n* = 3). (E, F) TRIM14-IκBα interaction was detected by co-immunoprecipitation (co-IP). (G) BV2 cells transfected with oeTRIM14 or empty vector were treated with 10 μM MG132 or vehicle (DMSO) for 4 h, followed by IκBα detection via Western blot. (H) oeTRIM14- or vector-transfected BV2 cells were incubated with 100 μg/mL CHX for indicated durations, and IκBα levels were analyzed by Western blot (*n* = 3). (I) IκBα ubiquitination was detected by IP assay. Data represent mean ± SD. *⁣*^*∗*^*p* < 0.05, *⁣*^*∗∗*^*p* < 0.01, *⁣*^*∗∗∗*^*p* < 0.001. CHX (cycloheximide), protein synthesis inhibitor; MG132, proteasome inhibitor; NC, negative control; oeTRIM14, TRIM14 overexpression; siTRIM14, siRNA targeting TRIM14.

As demonstrated by immunofluorescence (Figure 2A) and western blot (Figure 2B), TRIM14 knockdown significantly suppressed LPS-induced nuclear translocation of p65 in BV2 cells, indicating its essential role in NF-κB activation. Consistent with this observation, TRIM14 silencing increased IκBα protein levels upon LPS stimulation, while TRIM14 overexpression markedly reduced IκBα expression (Figure 2C). Co-IP experiments confirmed a direct physical interaction between TRIM14 and IκBα (Figure 2D), which was abolished upon TRIM14 knockdown (Figure 2E). Further mechanistic investigations revealed that TRIM14-mediated IκBα reduction was blocked by the proteasome inhibitor MG132 (Figure 2F). Additionally, TRIM14 overexpression accelerated IκBα degradation in cycloheximide (CHX) chase assays (0–12 h) (Figure 2G), and significantly enhanced IκBα ubiquitination (Figure 2H). These data collectively demonstrate that TRIM14 promotes ubiquitin-proteasome-dependent degradation of IκBα.

### 3.3. TRIM14 Promotes BV2 Cell M1 Polarization via the NF-κB Pathway

**Figure 3:**
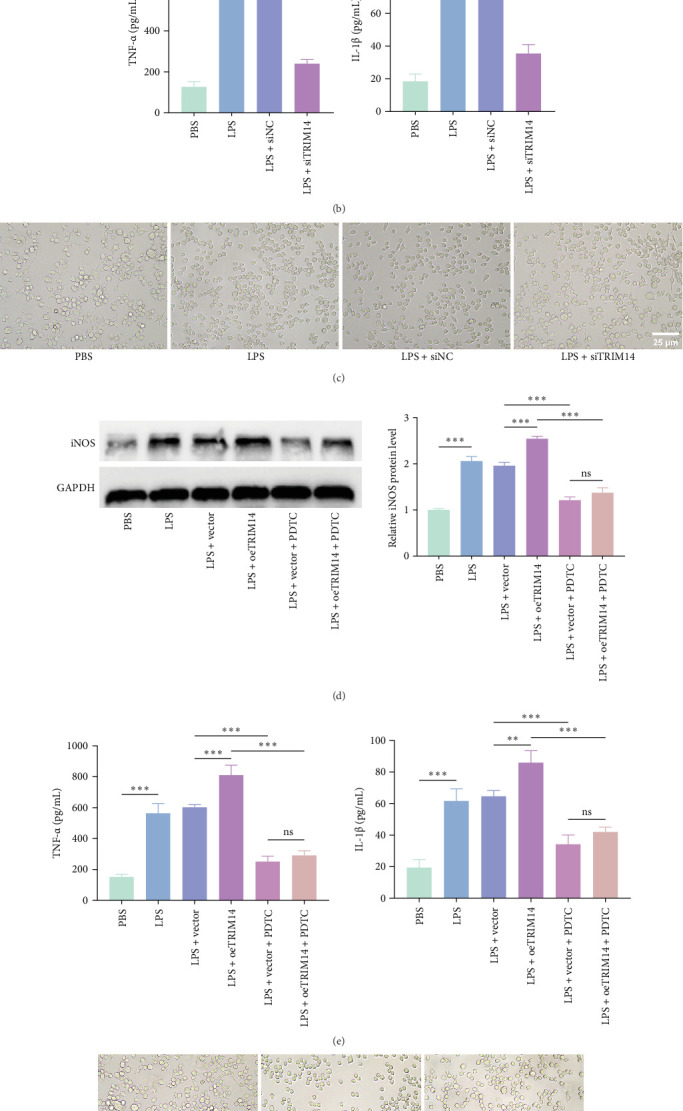
TRIM14 promotes M1 polarization of BV2 cells via the NF-κB pathway. After transfection with siTRIM14, BV2 cells were stimulated with LPS (100 ng/mL). (A) Western blot analysis was performed to detect iNOS protein expression in BV2 cells (*n* = 3). (B) Major inflammatory cytokine levels were measured by ELISA (*n* = 3). (C) Cell morphology was observed by light microscopy (scale bar: 25 μm). Representative images from three independent experiments are shown. BV2 cells were transfected with oeTRIM14 and pretreated with NF-κB inhibitor (PDTC, 100 μM) before LPS (100 ng/mL) stimulation. (D) iNOS expression was analyzed by western blot (*n* = 3). (E) Major inflammatory cytokine levels were measured by ELISA (*n* = 3). (F) Morphological changes of BV2 cells were observed under light microscopy (scale bar, 25 μm). Representative images were obtained from three independent experiments. Data represent mean ± SD. *⁣*^*∗*^*p* < 0.05, *⁣*^*∗∗*^*p* < 0.01, and *⁣*^*∗∗∗*^*p* < 0.001. NC, negative control. oeTRIM14, TRIM14 overexpression; siTRIM14, siRNA targeting TRIM14 overexpression.

As shown in Figure 3A, TRIM14 knockdown significantly attenuated the LPS-induced increase in iNOS protein expression (an M1 polarization marker) in BV2 cells. Furthermore, TRIM14 silencing reduced the secretion of proinflammatory cytokines TNF-α and IL-1β by M1-polarized microglia (Figure 3B). Morphological analysis revealed that TRIM14 knockdown markedly reversed the characteristic M1 polarization phenotype (cell rounding and process retraction) induced by LPS stimulation (Figure 3C). To substantiate the involvement of NF-κB signaling, we employed PDTC (a specific NF-κB inhibitor) in TRIM14-overexpressing cells. Notably, PDTC completely abolished TRIM14-mediated M1 polarization (Figure 3D–F), confirming the essential role of NF-κB pathway in this process.

### 3.4. TRIM14 Induces BV2 Cell Pyroptosis via the NF-κB Pathway

**Figure 4:**
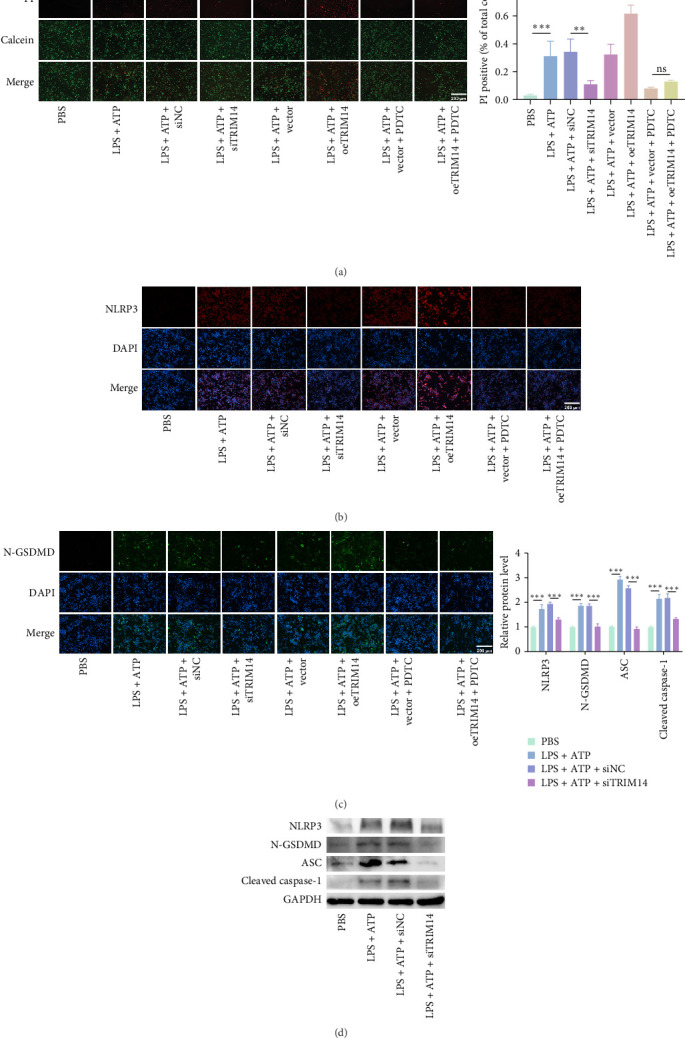
TRIM14 induces pyroptosis in BV2 cells via the NF-κB pathway. BV2 cells were transfected with siTRIM14 or oeTRIM14, pretreated with or without PDTC (NF-κB inhibitor, 100 μM), and then induced for pyroptosis with LPS (1 ng/mL) plus ATP (5 mM). (A) Double staining with PI (red) and calcein-AM (green). PI-positive cells were significantly reduced after PDTC pretreatment (scale bar: 200 μm; *n* = 3). (B, C) Fluorescence microscopy showed expression changes in different groups. NLRP3 and N-GSDMD were detected using fluor 594-conjugated (red) and FITC-conjugated (green) antibodies, respectively, with DAPI nuclear staining (blue; scale bar: 200 μm; *n* = 3). (D) Protein levels of NLRP3, N-GSDMD, ASC, and cleaved caspase-1 were analyzed by western blot (*n* = 3). Data represent mean ± SD. *⁣*^*∗∗*^*p* < 0.01 and *⁣*^*∗∗∗*^*p* < 0.001. ATP, adenosine triphosphate; NC, negative control. oeTRIM14, TRIM14 overexpression; siTRIM14, siRNA targeting TRIM14.

Using calcein AM/PI costaining to assess membrane integrity (a hallmark of pyroptosis), we found that TRIM14 knockdown significantly reduced LPS + ATP-induced BV2 cell pyroptosis, while TRIM14 overexpression markedly enhanced this process (Figure 4A). Notably, the NF-κB inhibitor PDTC effectively reversed TRIM14-mediated pyroptotic cell death. At the molecular level, TRIM14 silencing suppressed the upregulation of pyroptosis-related proteins (NLRP3, N-GSDMD, ASC, and cleaved caspase-1) in LPS + ATP-stimulated BV2 cells (Figure 4D). Immunofluorescence analysis further confirmed these findings, demonstrating that PDTC treatment abolished TRIM14-induced accumulation of NLRP3 and N-GSDMD (Figure 4B, C), consistent with the western blot results. Collectively, these data demonstrate that TRIM14 promotes BV2 cell pyroptosis through NF-κB-dependent regulation of the NLRP3 inflammasome pathway.

### 3.5. Effects of BV2 Cell Groups on Astrocytes and Neurons

**Figure 5:**
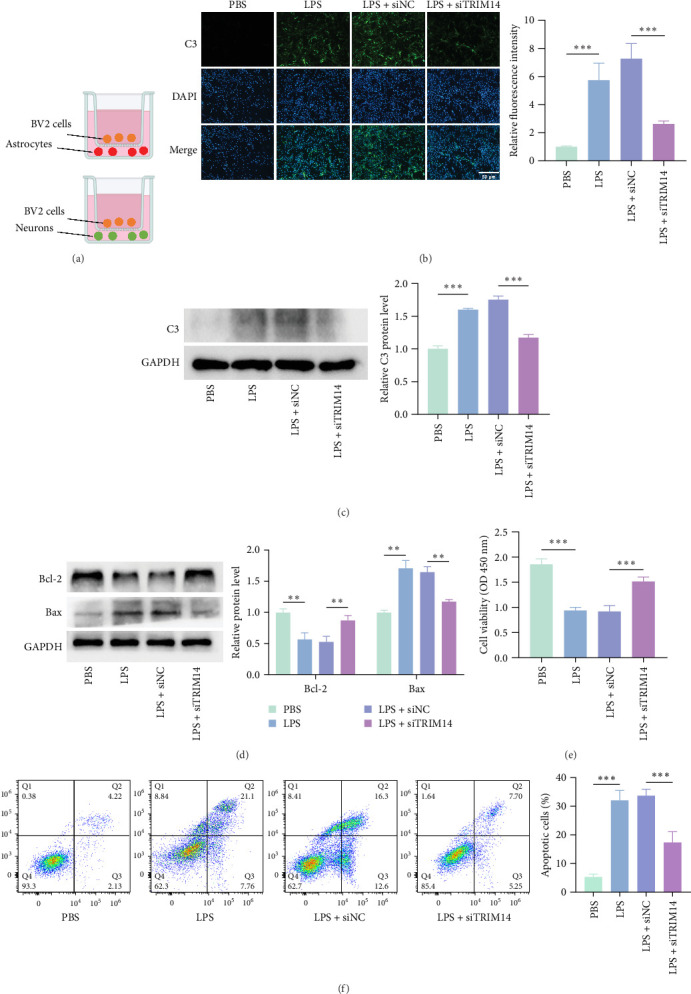
Inhibition of TRIM14 alleviates BV2 cell-mediated inflammatory stimulation to astrocytes and neurons. (A) BV2 cells transfected with siTRIM14 or siNC were stimulated with LPS (100 ng/mL), then cocultured with astrocytes and neurons in a Transwell system. (B) C3 expression in astrocytes was visualized by fluorescence microscopy using FITC-conjugated antibody (green) with DAPI nuclear counterstain (blue; scale bar: 50 μm; *n* = 3). (C) C3 protein levels in astrocytes were quantified by western blot (*n* = 3). (D) Neuronal Bcl-2 and Bax protein levels were analyzed by western blot (*n* = 3). (E) Neuronal viability was assessed by CCK-8 assay (*n* = 3). (F) Neuronal apoptosis rates were determined by flow cytometry (*n* = 3). Data represent mean ± SD. *⁣*^*∗∗*^*p* < 0.01 and *⁣*^*∗∗∗*^*p* < 0.001. NC, negative control. siTRIM14, siRNA targeting TRIM14.

BV2 cells subjected to different treatments were cocultured with astrocytes or neurons using a Transwell system (Figure 5A). Immunofluorescence (Figure 5B) and western blot (Figure 5C) analyses revealed that astrocytes cocultured with TRIM14-knockdown BV2 cells exhibited significantly reduced levels of C3, a marker for A1 reactive astrocytes. In neurons, coculture with TRIM14-silenced BV2 cells resulted in decreased expression of the proapoptotic protein Bax and increased levels of the antiapoptotic protein Bcl-2 (Figure 5D). Consistently, these neurons showed enhanced viability (Figure 5E) and reduced apoptosis rates (Figure 5F). These findings demonstrate that TRIM14 knockdown in BV2 cells attenuates their neuroinflammatory effects on both astrocytes and neurons following LPS stimulation.

### 3.6. TRIM14 Inhibition Suppresses M1 Polarization and Pyroptosis of Microglia in the Lesion Area

**Figure 6:**
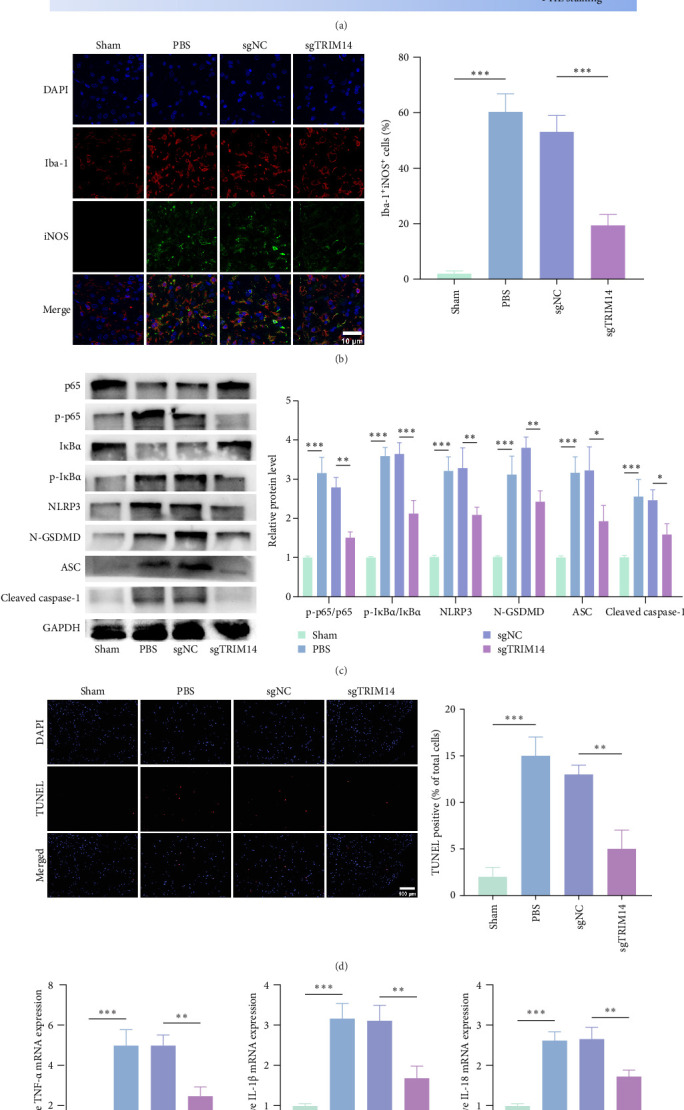
Knockdown of TRIM14 inhibits M1 polarization and pyroptosis of microglia in rat spinal cord. (A) Schematic of the in vivo experimental design. (B) Immunofluorescence images showing M1 microglia distribution at injury sites (7 dpi). Bar graph quantifies iNOS^+^Iba-1^+^/Iba-1^+^ cell ratios (scale bar: 10 μm; *n* = 3). (C) Protein expression in injured spinal cords (7 dpi) analyzed by western blot (*n* = 3). (D) Representative TUNEL staining (7 dpi; scale bar: 100 μm; *n* = 3). Bar graph shows TUNEL^+^ cell ratios. (E) Inflammatory factor mRNA levels (*n* = 3). Data represent mean ± SD. *⁣*^*∗*^*p* < 0.05, *⁣*^*∗∗*^*p* < 0.01, and *⁣*^*∗∗∗*^*p* < 0.001. NC, negative control. sgTRIM14, sgRNA targeting TRIM14.

Figure 6A shows a schematic of the in vivo experimental procedure. To characterize microglial phenotypes at the lesion site, we performed immunofluorescence staining for Iba-1 (microglial marker) and iNOS (M1 polarization marker) in spinal cord tissues at 7 dpi. As shown in Figure 6B, TRIM14 knockdown significantly reduced the proportion of Iba-1^+^/iNOS^+^ cells in the injured area. Western blot analysis revealed that TRIM14 inhibition markedly decreased the ratios of p-p65/p65 and p-IκBα/IκBα (Figure 6C), indicating suppression of NF-κB signaling. Consistent with this finding, qPCR analysis demonstrated reduced mRNA expression of M1-derived proinflammatory cytokines (TNF-α and IL-1β) in the TRIM14 knockdown group (Figure 6E). Furthermore, TRIM14 suppression attenuated pyroptosis as evidenced by decreased protein levels of NLRP3 inflammasome components including N-GSDMD, ASC, and cleaved caspase-1 (Figure 6C), along with reduced mRNA expression of IL-1β and IL-18 (Figure 6E). Collectively, these data demonstrate that TRIM14 knockdown in spinal microglia effectively mitigates both M1 polarization and pyroptosis after SCI.

### 3.7. TRIM14 Inhibition Promotes Axon Regrowth and Neural Survival After SCI

**Figure 7:**
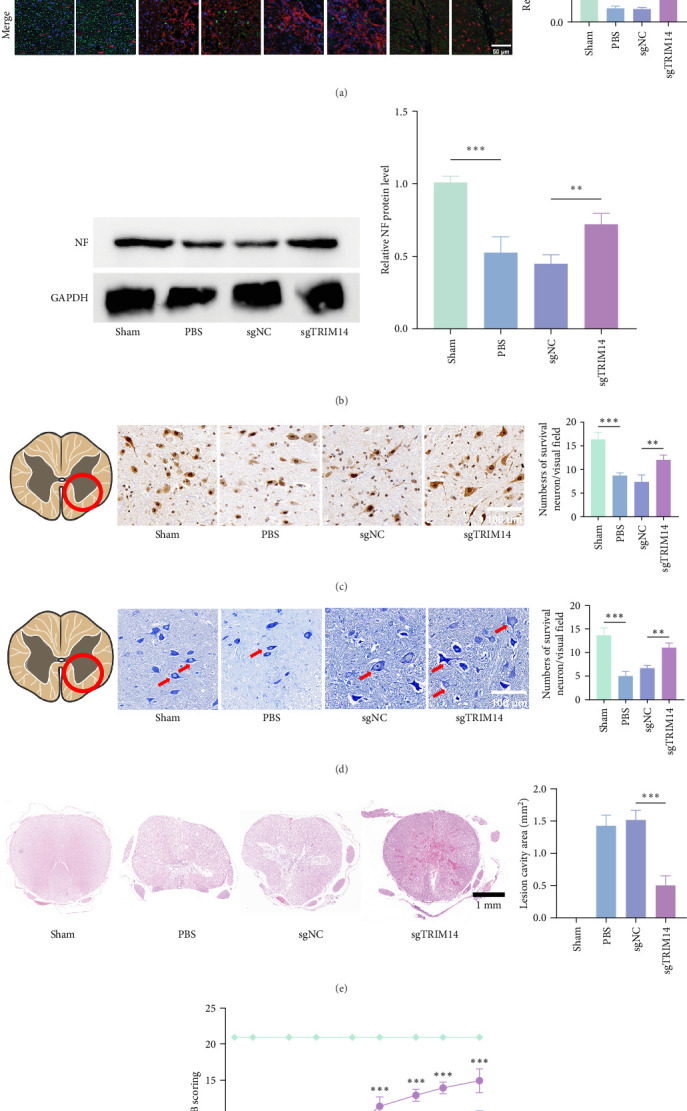
TRIM14 knockdown promotes neuronal survival, spinal tissue repair, and functional recovery after spinal cord injury in rats. (A) Representative immunofluorescence images showing NF (green, neurofilament) and GFAP (red, glial fibrillary acidic protein) distribution in spinal cord lesions of rats from different groups at 28 days post-injury (dpi). Scale bars: 500 μm (low magnification), 50 μm (high magnification); *n* = 3. Quantitative analysis of mean fluorescence density for NF and GFAP at the injury epicenter is shown (*n* = 3). (B) Protein levels of NF in spinal cord tissues analyzed by western blot (*n* = 3). (C) Representative IHC images of NeuN^+^ neurons in injured spinal cords (28 dpi; scale bar: 100 μm; *n* = 3). Bar graph indicates the mean number of surviving NeuN^+^ neurons per field. (D) Nissl staining of injured spinal cords (28 dpi; scale bar: 100 μm; *n* = 3). Arrows denote Nissl-positive cells. Bar graph displays the mean number of Nissl^+^ cells per field. In (C, D), three stained sections were randomly selected from each experimental rats to evaluate the average number of surviving neurons in the anterior horns of spinal cord. (E) HE staining of spinal cord lesion cavities. Bar graph quantifies lesion cavity areas across groups (*n* = 3). (F) Hindlimb motor function evaluated by Basso–Beattie–Bresnahan (BBB) locomotor rating scale (*n* = 3). Interrater reliability was confirmed by an intraclass correlation coefficient (ICC > 0.85). Data represent mean ± SD. *⁣*^*∗*^*p* < 0.05, *⁣*^*∗∗*^*p* < 0.01, and *⁣*^*∗∗∗*^*p* < 0.001. NC, negative control. sgTRIM14, sgRNA targeting TRIM14.

We evaluated the therapeutic effects of TRIM14 inhibition. Our findings demonstrated a higher fluorescence density of NF and a lower fluorescence density of GFAP in the sgTRIM14-treated group at 28 dpi (Figure 7A). IHC staining of NeuN revealed an increased numbers of surviving neurons in the spinal cord of sgTRIM14-treated group at 28 dpi (Figure 7C). Nissl staining images demonstrated an increase in the number of Nissl-positive cells in the sgTRIM14-treated group at 28 dpi (Figure 7D). The TUNEL staining results at 7 dpi indicated a reduction in nerve cell apoptosis rate in the sgTRIM14-treated group (Figure 6D). Additionally, western blot analysis revealed significantly increased expression of NF protein in the sgTRIM14 group. These findings suggest that the environment induced by TRIM14 inhibition promotes axon regrowth and contributes to neural survival after SCI.

### 3.8. TRIM14 Inhibition Promotes Tissue Repair and Motor Functional Recovery After SCI in Rats

Histopathological changes in the injured spinal cord were assessed by HE staining at 28 dpi following sgTRIM14 treatment. Compared to the PBS and sgControl groups, which exhibited extensive lesion areas, sgTRIM14 administration significantly reduced the lesion size (Figure 7E). Functional recovery was evaluated using BBB scoring. All groups except the sham group showed initial scores of 0 post-SCI, confirming successful model establishment. While spontaneous hindlimb functional recovery was observed in all groups, the sgTRIM14-treated group demonstrated significantly higher BBB scores than the sgControl group at 14, 21, and 28 dpi (Figure 7F).

## 4. Discussion

The intricate pathophysiology of SCI unfolds through a devastating interplay of primary mechanical damage and secondary biochemical cascades [18, 19], with neuroinflammation emerging as a central orchestrator of tissue degeneration and failed regeneration [20, 21]. Within this inflammatory milieu, microglia—the resident immune sentinels of the CNS—transition from surveillant protectors to destructive aggressors, adopting a proinflammatory M1 phenotype that unleashes cytotoxic cytokines and propagates pyroptotic cell death [22–24]. Our study illuminates TRIM14 as a master regulator of this pathological transformation, bridging NF-κB-driven polarization to NLRP3-mediated pyroptosis through a mechanism rooted in ubiquitination-dependent signaling. By dissecting TRIM14's dual role in coordinating these processes, we not only redefine the molecular hierarchy of neuroinflammatory cascades in SCI but also unveil a therapeutic strategy with transformative potential for CNS repair.

TRIMs are RING domain-containing E3 ligases involved in the regulation of cellular homeostasis, metabolism, cell death, inflammation, and host defense [25–27]. Current research on TRIM14 has primarily focused on its roles in tumorigenesis and acute inflammatory responses [14, 28–30], with demonstrated involvement in promoting psoriasis and osteoarthritis progression [13, 31]. However, its functions in CNS remain largely unexplored. Prior work has demonstrated that TRIM14 inhibition alleviates neuronal apoptosis in ischemic brain injury by suppressing neuroinflammation [32]. Given the shared neuroinflammatory mechanisms between SCI and cerebral ischemia, we hypothesized that TRIM14 may similarly play a pivotal role in SCI pathogenesis. Substantiating this hypothesis, we observed marked upregulation of TRIM14 in lesioned rat spinal cord tissue compared to sham controls, positioning TRIM14 as a pivotal regulator of post-SCI inflammatory cascades.

TRIM14 has been characterized as a positive regulator of the NF-κB pathway [12, 33, 34], which undergoes pronounced activation following SCI. Targeted inhibition of NF-κB signaling has demonstrated therapeutic potential in mitigating post-SCI inflammation [35–37], prompting our focus on the TRIM14/NF-κB axis as a key investigative pathway. After SCI, microglia rapidly infiltrate the injury epicenter and adopt a pro-inflammatory M1 phenotype characterized by TNF-α and IL-1β secretion, thereby initiating downstream inflammatory cascades [3, 38, 39]. Although NF-κB is recognized as a master regulator of microglial M1 polarization [40, 41], TRIM14's role in this process remained unexplored. Our preliminary screening revealed significant TRIM14 upregulation in activated microglia, establishing microglia as the cellular focus of this study. Mechanistically, TRIM14 knockdown attenuated LPS-induced M1 polarization in BV2 cells, as evidenced by reduced iNOS expression. Conversely, TRIM14 overexpression exacerbated M1 polarization—a phenotype completely reversed by PDTC-mediated NF-κB inhibition. These findings definitively position TRIM14 upstream of NF-κB in regulating microglial M1 reprograming. While Zhang et al. [42] demonstrated that NF-κB inhibition drives microglial repolarization from M1 to M2 phenotypes, our contrasting findings suggest that M1/M2 switching may require coordinated regulation by additional pathways—such as MAPK and JAK/STAT signaling axes, which are previously implicated in microglial plasticity—beyond canonical NF-κB signaling [43, 44].

We next sought to delineate the precise mechanism by which TRIM14 activates the NF-κB pathway. While Chen et al. [12] reported TRIM14-mediated non-canonical NF-κB activation via the p100/p52 axis in colorectal cancer, and Li et al. [13] demonstrated its role in promoting TRAF3 autophagic degradation to activate NF-κB in psoriasis, our study revealed a distinct mechanism. Intriguingly, TRIM14 knockdown or overexpression caused pronounced IκBα fluctuations in microglia, prompting us to hypothesize direct TRIM14-IκBα interaction. Subsequent co-IP assays confirmed their physical binding. As an E3 ubiquitin ligase, TRIM14 facilitated IκBα ubiquitination and proteasomal degradation, as evidenced by CHX chase assays and in vitro ubiquitination reconstitution experiments (Figure 2). This defines an unreported mechanism of NF-κB regulation by TRIM14 in the CNS. Notably, whether IκBα ubiquitination represents the sole regulatory mechanism for TRIM14-mediated NF-κB activation during microglial polarization remains unaddressed—a critical knowledge gap that warrants systematic exploration through multiomics approaches or domain-specific mutagenesis.

Following SCI, the NLRP3 inflammasome is predominantly activated in microglia, where caspase-1-mediated cleavage promotes the extracellular release of IL-1β and IL-18, thereby triggering a cascade of inflammatory responses [45, 46]. Accumulating evidence has established the NF-κB pathway as a key upstream regulator of NLRP3 inflammasome activation [47, 48]. Building on this, we further investigated the role of TRIM14 in microglial pyroptosis. Our data further demonstrate that TRIM14 exerts promotional effects on microglial pyroptosis through NF-κB pathway modulation. Mechanistically, TRIM14 overexpression enhanced caspase-1 activation and GSDMD cleavage (key pyroptotic executors), whereas pharmacological NF-κB inhibition (PDTC) reversed these effects, positioning NF-κB as the central mediator of TRIM14's pro-pyroptotic function (Figure 4).

Following SCI, the crosstalk between glial cells and neurons is critical for maintaining local microenvironmental homeostasis [49, 50]. To investigate whether TRIM14-knockdown BV2 microglia mitigate inflammatory stimulation to astrocytes and neurons, we established an in vitro coculture system. Our results demonstrated that astrocytes cocultured with TRIM14-deficient BV2 cells exhibited reduced activation (as indicated by decreased C3 expression). Furthermore, Neurons cocultured with TRIM14-knockdown BV2 cells exhibited significantly reduced apoptosis. These findings suggest that TRIM14 knockdown in microglia attenuates neuroinflammatory crosstalk, thereby protecting both astrocytes and neurons from secondary injury.

For in vivo validation, we established a rat SCI model and employed intralesional AAV-CRISPR/CasRx delivery to achieve TRIM14 knockdown within the injury microenvironment. To account for the temporal requirements of viral transduction efficiency and the acute neuroinflammatory phase post-SCI, spinal cord tissues were harvested at 7 dpi for assessing microglial polarization and pyroptosis in the lesion epicenter [51]. Our results demonstrate that TRIM14 inhibition attenuates both M1 polarization and pyroptosis of microglia at the lesion site. The paramount objective of SCI therapeutic intervention lies in promoting neuronal survival, axonal regeneration, and neural circuit reorganization. This neuroprotective imperative motivated our focused evaluation of glial scar dynamics—a major barrier to axonal regeneration—and neuronal integrity at the lesion epicenter. Spinal cord tissues were harvested at 28 dpi for comprehensive analyses [52]. Combined GFAP/NF immunofluorescence and western blotting demonstrated that TRIM14 knockdown attenuated glial scar formation while promoting axonal regeneration. Consistent with these findings, IHC, Nissl staining, and TUNEL assays revealed enhanced neuronal survival in TRIM14-deficient groups, with fewer apoptotic neurons and higher Nissl body density compared to controls. HE staining further corroborated tissue preservation, showing a reduction in cystic cavity volume. Functional assessment using the BBB locomotor scale (0–21) demonstrated significant motor recovery in TRIM14-knockdown rats from 14 dpi onward.

The improved functional recovery and neural repair observed after TRIM14 knockdown are likely the result of a synergistic effect, primarily driven by an indirect mechanism. The initial and crucial effect is the attenuation of secondary damage by creating a less hostile microenvironment. By suppressing microglial M1 polarization and pyroptosis, TRIM14 inhibition significantly reduces the release of pro-inflammatory cytokines (e.g., IL-1β and TNF-α) and prevents the spread of inflammatory cell death, thereby indirectly promoting neuronal survival and axonal regeneration [53]. While a direct effect on neurons cannot be entirely ruled out, our in vitro Transwell coculture data suggest that the beneficial outcomes are predominantly mediated through modulating the microglial secretome, which in turn exerts a paracrine effect on neighboring neurons and astrocytes.

From a translational perspective, our study nominates TRIM14 as a promising therapeutic target for SCI. However, translating this finding into a clinical strategy faces several challenges. The primary hurdle likely lies in the efficient and targeted delivery of a TRIM14 inhibitor (e.g., siRNA and small molecule) to the spinal cord lesion site, potentially requiring advanced delivery systems like nanoparticles or engineered viral vectors [54]. Furthermore, determining the optimal therapeutic time window post-injury to effectively intervene in the neuroinflammatory cascade without disrupting potential beneficial aspects of microglial function will be critical for future preclinical development [55].

Furthermore, the pathological role of the TRIM14/NF-κB/NLRP3 axis we identified may not be limited to SCI. Neuroinflammation and microglial dysregulation are central features of many chronic neurodegenerative diseases, such as Alzheimer's disease and Parkinson's disease, where NLRP3-mediated pyroptosis has also been implicated [56]. Therefore, targeting TRIM14 could represent a versatile therapeutic strategy for a broader spectrum of neuroinflammatory and neurodegenerative conditions, warranting further investigation in these contexts.

While our findings establish TRIM14 as a key player in SCI, several questions remain unresolved. First, the long-term consequences of TRIM14 suppression require scrutiny. Chronic inhibition of ubiquitination pathways may impair microglial surveillance or synaptic pruning—processes vital for CNS homeostasis. Longitudinal studies tracking immune competence and cognitive function in TRIM14-silenced animals will address these concerns. In addition, the translational leap from rodents to humans demands rigorous validation. Species-specific differences in TRIM14 regulation could influence therapeutic efficacy.

In conclusion, our work expands the functional repertoire of TRIM proteins in CNS disorders. This study redefines TRIM14 as a molecular fulcrum in SCI pathogenesis, orchestrating microglial polarization and pyroptosis through a ubiquitination-dependent NF-κB/NLRP3 axis. By disentangling this intricate signaling network, we provide a blueprint for precision interventions that reconcile immune modulation with neural repair. As the field pivots toward combinatorial and cell type-specific therapies, TRIM14 inhibition emerges as a cornerstone strategy—one that may ultimately transform the bleak prognosis of SCI into a narrative of hope and recovery.

## 5. Conclusions

This study identifies TRIM14 as a critical pathogenic regulator of neuroinflammation in SCI, where its upregulation exacerbates secondary damage by orchestrating microglial M1 polarization and NLRP3-mediated pyroptosis through ubiquitination-dependent activation of the NF-κB pathway. Mechanistically, TRIM14 directly binds IκBα, triggering its ubiquitin-proteasomal degradation to liberate NF-κB for nuclear translocation and subsequent NLRP3 inflammasome assembly—culminating in GSDMD-driven pyroptotic cell death and proinflammatory cytokine storms. Crucially, in vivoAAV-CRISPR/CasRx-mediated silencing of TRIM14 in SCI rats suppressed this neuroinflammatory cascade, attenuating neuronal apoptosis and astrocytic reactivity while enhancing axonal regeneration and tissue preservation. These cellular reparative effects translated to significant functional recovery, as evidenced by improved locomotor scores and reduced lesion pathology. Our work thus delineates the TRIM14-IκBα-NF-κB-NLRP3 axis as a previously unrecognized signaling nexus in SCI, positioning TRIM14 inhibition as a promising therapeutic strategy to recalibrate the injury microenvironment from destructive inflammation toward neural repair, with direct implications for treating CNS trauma.

## Data Availability

The data that support the findings of this study are available on request from the corresponding author. The data are not publicly available due to privacy or ethical restrictions.
